# Supplementary material for: DNA immunotherapy targeting BARF1 induces potent anti-tumor responses against Epstein-Barr-virus-associated carcinomas
Source: Mol Ther Oncolytics. 2021 Dec 21;24:218–29. doi: 10.1016/j.omto.2021.12.017 (PMC8761958; doi:10.1016/j.omto.2021.12.017)
Supplement: Document S1. Figures S1–S3 [file mmc1.pdf]

OMTO, Volume *24*

Supplemental information

DNA immunotherapy targeting BARF1 induces potent anti-tumor responses against Epstein-Barr-virus-associated carcinomas

Xizhou Zhu, Alfredo Perales-Puchalt, Krzysztof Wojtak, Ziyang Xu, Kun Yun, Pratik S. Bhojnagarwala, Devivasha Bordoloi, Daniel H. Park, Kevin Liaw, Mamadou A. Bah, Paul M. Lieberman, Ebony N. Gary, Ami Patel, and David B. Weiner

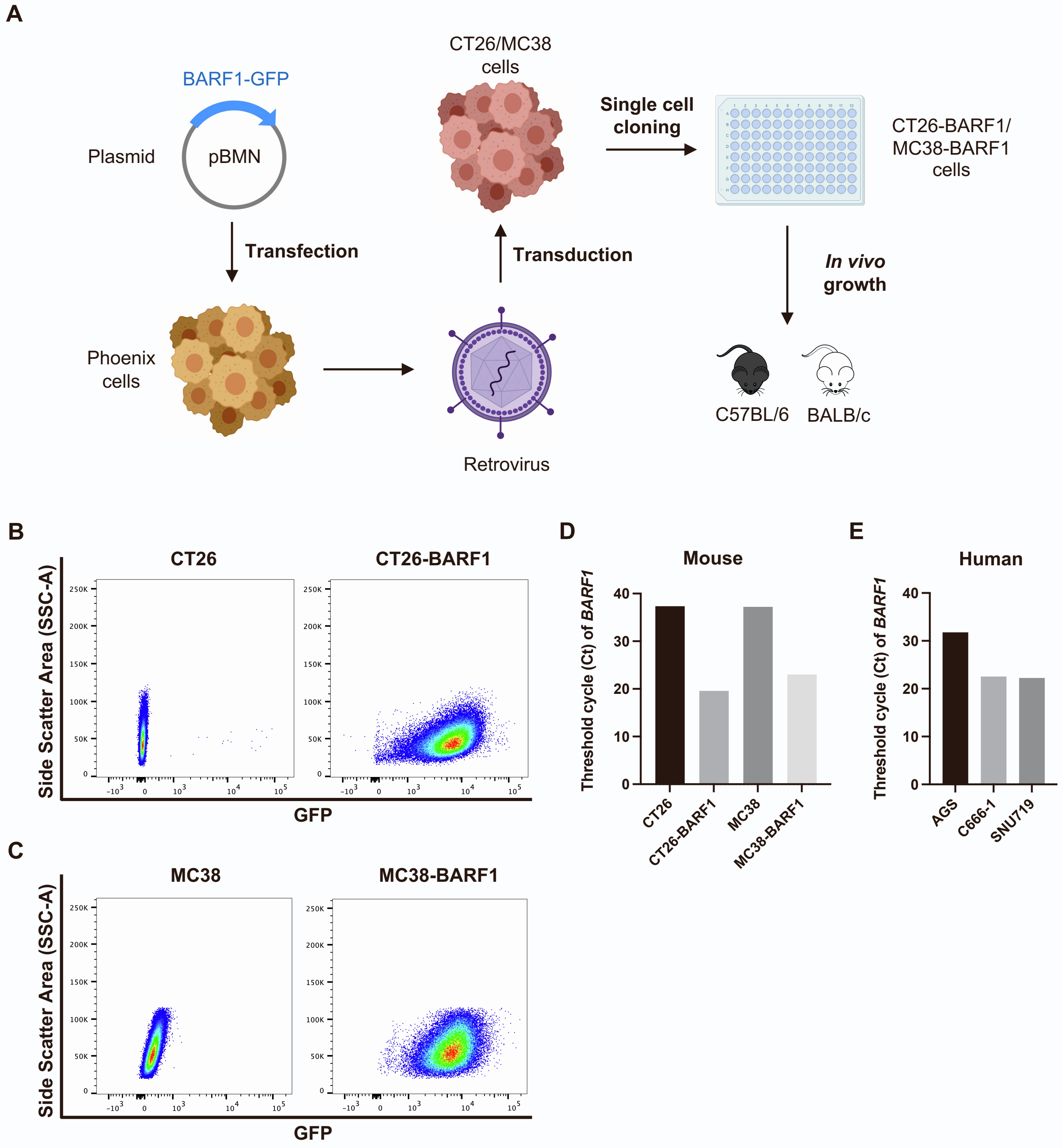

Supplemental Figure 1. Generation and validation of BARF1⁺ tumor models. (A) Workflow of retroviral transduction and single-cell cloning of CT26-BARF1 and MC38-BARF1 cells. (B and C) Clonality of CT26-BARF1 (B) and MC38-BARF1 (C) cell lines, after single-cell cloning, shown by GFP expression through flow cytometry. (D and E) Threshold cycles of *BARF1* gene amplification by RT-qPCR in transduced and parental mouse cancer cell lines (D) and human EBV positive (C666-1 and SUN719) and negative (AGS) cancer cell lines (E).

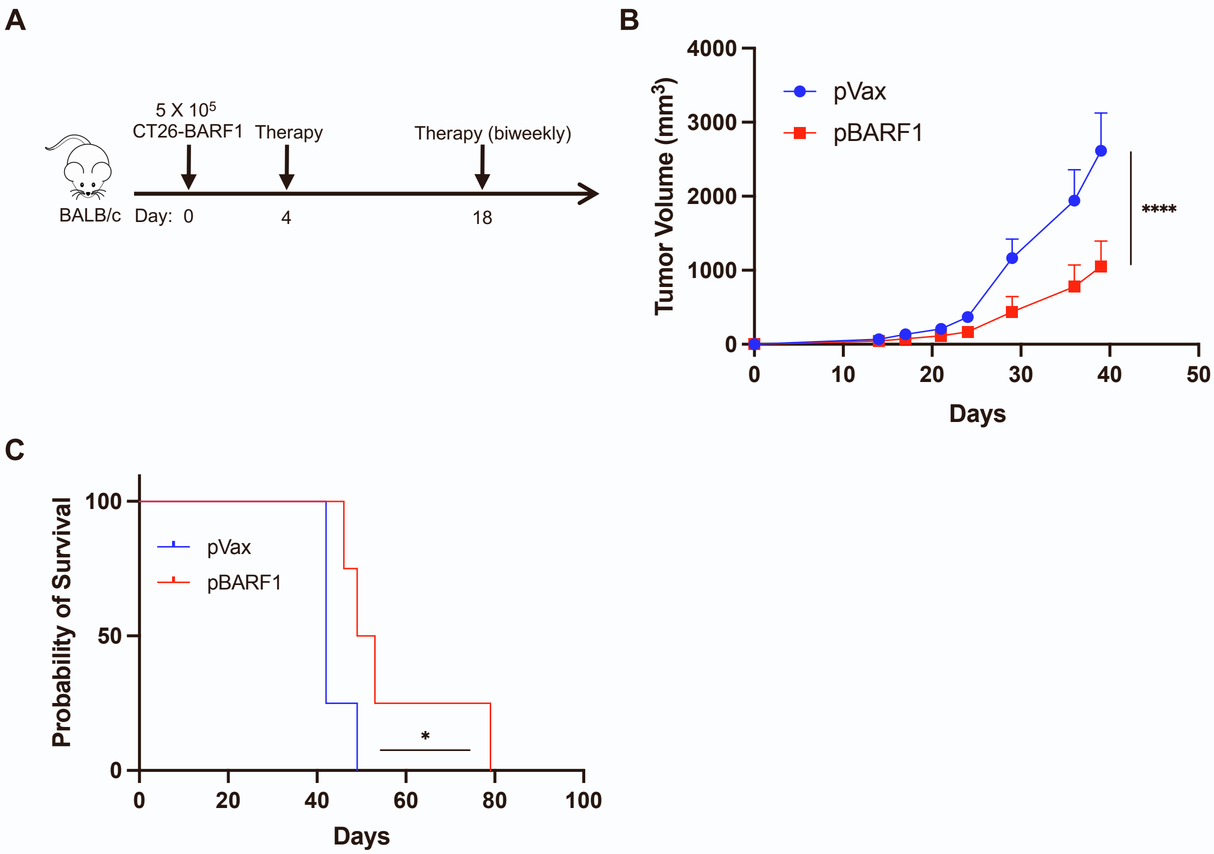

Supplemental Figure 2. pBARF1 improves survival in the therapeutic tumor model in BALB/c mice. (A) Study outline for the therapeutic tumor model. The mice were injected with CT26-BARF1 cells and immunized with pBARF1 biweekly starting on day 4. (B) Tumor volume measurements of the study described in (A). (C) Survival curve of the study described in (A). Significance for tumor volume was determined by two-way ANOVA. Significance for survival was determined by the log-rank test. *$p < 0.05$, ****$p < 0.0001$. $n = 4$ mice/group. Error bars indicate mean ± SEM.

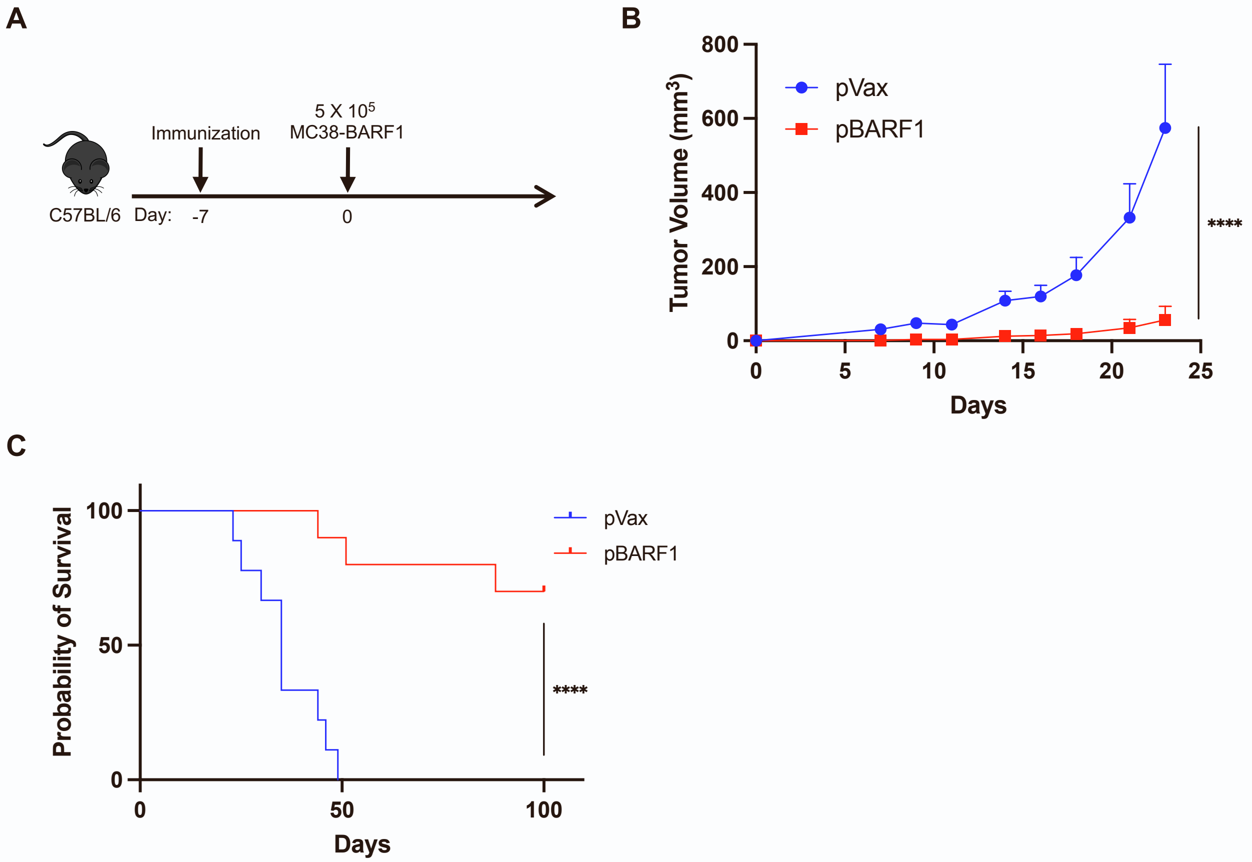

Supplemental Figure 3. Single immunization of pBARF1 prevents tumor progression in C57BL/6 mice. (A) Study outline for the pre-challenge immunization model. The mice were immunized with one dose of pBARF1 and injected with MC38-BARF1 cells one week after immunization. (B) Tumor volume measurements of the study described in (A). (C) Survival curve of the study described in (A). Significance for tumor volume was determined by two-way ANOVA. Significance for survival was determined by the log-rank test. ****$p < 0.0001$. $n = 10$ mice/group. Error bars indicate mean ± SEM.
