# Supplementary material for: DNA immunotherapy targeting BARF1 induces potent anti-tumor responses against Epstein-Barr-virus-associated carcinomas
Source: Mol Ther Oncolytics. 2021 Dec 21;24:218–29. doi: 10.1016/j.omto.2021.12.017 (PMC8761958; doi:10.1016/j.omto.2021.12.017)
Supplement: Document S2. Article plus supplemental information [file mmc2.pdf]

Molecular Therapy
Oncolytics
Original Article

DNA immunotherapy targeting BARF1 induces potent anti-tumor responses against Epstein-Barr-virus-associated carcinomas

Xizhou Zhu,[1] Alfredo Perales-Puchalt,[1] Krzysztof Wojtak,[1] Ziyang Xu,[1] Kun Yun,[1] Pratik S. Bhojnagarwala,[1] Devivasha Bordoloi,[1] Daniel H. Park,[1] Kevin Liaw,[1] Mamadou A. Bah,[1] Paul M. Lieberman,[2] Ebony N. Gary,[1] Ami Patel,[1] and David B. Weiner[1]

[1]Vaccine and Immunotherapy Center, The Wistar Institute, 3601 Spruce Street, Philadelphia, PA 19104, USA; [2]Gene Expression and Regulation Program, The Wistar Institute, Philadelphia, PA 19104, USA

Latent Epstein-Barr virus (EBV) infection is associated with several types of cancer. Several clinical studies have targeted EBV antigens as immune therapeutic targets with limited efficacy of EBV malignancies, suggesting that additional targets might be important. *Bam*HI-A rightward frame 1 (BARF1) is an EBV antigen that is highly expressed in EBV$^+$ nasopharyngeal carcinoma (NPC) and EBV-associated gastric carcinoma (EBVaGC). BARF1 antigen can transform human epithelial cells *in vivo*. BARF1-specific antibodies and cytotoxic T cells were detected in some EBV$^+$ NPC patients. However, BARF1 has not been evaluated as an antigen in the context of therapeutic immunization. Its possible importance in this context is unclear. Here, we developed a synthetic-DNA-based expression cassette as immunotherapy targeting BARF1 (pBARF1). Immunization with pBARF1 induced potent antigen-specific humoral and T cell responses *in vivo*. Immunization with pBARF1 plasmid impacted tumor progression through the induction of CD8$^+$ T cells in novel BARF1$^+$ carcinoma models. Using an *in vivo* imaging system, we observed that pBARF1-immunized animals rapidly cleared cancer cells. We demonstrated that pBARF1 can induce antigen-specific immune responses that can impact cancer progression. Further study of this immune target is likely important as part of therapeutic approaches for EBV$^+$ malignancies.

INTRODUCTION

Epstein-Barr virus (EBV), also known as human gammaherpesvirus 4 (HHV-4), is highly ubiquitous, with more than 95% of the world's population infected by EBV.[1] As the first identified oncogenic virus, EBV is associated with several types of lymphomas and carcinomas, including nasopharyngeal carcinoma (NPC) and EBV-associated gastric carcinoma (EBVaGC).[2] NPC and EBVaGC account for more than 92% of all EBV-associated cancers, resulting in approximately 160,000 cases per year globally.[3] The majority of NPCs are EBV$^+$, exhibiting type III viral latency associated with the expression of latent membrane proteins (LMP1 and LMP2) and EBV nuclear antigen (EBNA1), as well as EBV *Bam*HI-A region rightward transcripts (BARTs).[3] Early and locally advanced cancer responds well to radiation or concurrent chemoradiation therapies. However, treatment for recurrent or metastatic disease is limited, and the prognosis is poor.[4] EBVaGC accounts for about 9% of all gastric cancers (GCs) and displays a unique molecular signature compared with other GC subtypes, including showing unique DNA hypermethylation as well as upregulation of programmed death ligands 1 and 2 (PD-L1/2).[5] While there have been many studies, there is currently no EBV-targeted immune therapy approved for NPC or EBVaGC.

Immunotherapy is becoming a foundational approach for treating diverse cancers. Therapies including allogeneic T cell transfer, targeted antibodies, and therapeutic immunizations have been explored in preclinical and clinical studies for NPC and EBVaGC.[6–9] Pembrolizumab, a PD-1 inhibitor, was approved by the FDA for recurrent or metastatic NPC.[10] However, the overall response rates are limited to 26.3% of patients, with a median progression-free survival (PFS) of just 6.5 months. The development of new approaches and immunotherapy targets remain important. Previous studies focused on targeting EBV predominate latency antigens, particularly EBNA1, LMP1, and LMP2, as potential therapeutic targets. These remain under study, but alone, they have not individually demonstrated enough control or clearance of EBV disease.[9,11] Another less-studied target, *Bam*HI-A rightward frame 1 (BARF1), is an EBV protein that is highly expressed in NPC and EBVaGC.[12,13] BARF1 is 221 amino acids in length and contains two immunoglobulin-like domains.[14] It is cleaved after the first 20 amino acids and secreted as a self-assembling hexamer (sBARF1). BARF1 contains interaction sites that allow it to bind to human macrophage colony stimulating factor (M-CSF) through its N-terminal domain and to human M-CSF-receptor homologous regions located in its C-terminal domain. Structural studies have shown that sBARF1 can interfere with monocyte differentiation

Received 25 July 2021; accepted 17 December 2021;
https://doi.org/10.1016/j.omto.2021.12.017.

Correspondence: David B. Weiner, PhD, Vaccine and Immunotherapy Center, The Wistar Institute, 3601 Spruce Street, Philadelphia, PA 19104, USA.
E-mail: dweiner@wistar.org

 © 2022 The Authors.
This is an open access article under the CC BY license (http://creativecommons.org/licenses/by/4.0/).

through binding to M-CSF and acting as a decoy receptor.[15] This interaction can reduce the expression of markers for macrophage differentiation such as CD11b, CD14, CD16, and CD169 and inhibit the production of interferon-alpha (IFN-α) by mononuclear cells, which is an important component of the host anti-viral immune response.[14]

Other oncogenic effects of BARF1 include promoting cell proliferation, inducing cell immortalization, and anti-apoptosis.[16,17] Importantly, previous studies have demonstrated BARF1 to be immunogenic, as BARF1-specific antibodies and T cells were detected in some EBV$^+$ NPC patients.[18,19] Reports also showed that T cells specific to BARF1 epitopes, expanded from patient blood samples *ex vivo*, are able to kill BARF1$^+$ cancer cells.[19,20] Thus, BARF1 appears to be an interesting candidate to be further studied for targeting EBV-associated cancer.

Most immunotherapies described for EBV-associated diseases have focused on adoptive cell transfer (ACT) and therapeutic immunization.[21] For the ACT strategy, EBV-specific cytotoxic T lymphocytes (CTLs) were generated *in vitro* by using EBV-transformed lymphoblastoid cell lines (LCLs) as antigen-presenting cells (APCs). Adoptive transfer of CTLs targeting EBNA1, LMP1, and LMP2 has shown some level of anti-tumor responses in some NPC patients.[7,22] In therapeutic immunization approaches, different approaches displaying different EBV antigens have been studied in clinical and preclinical studies. These include autologous dendritic cells pulsed with human leukocyte antigen (HLA)-restricted epitope peptides from LMP2, recombinant vaccinia virus encoding an EBNA1/LMP2 fusion protein, and a recombinant adenoviral vector expressing the LMP2 antigen.[9,23,24] These studies have shown modest efficacy, suggesting that additional antigens might be important for improving their impact.[21,25] Additional EBV viral targets might provide immune breadth or potency, which could improve immunotherapeutic efficacy. In this regard, the BARF1 antigen of EBV is interesting; however, this target has not been studied for its potential in therapeutic immunization approaches.

Here, we developed an optimized immunogen encoding the EBV antigen BARF1 as a synthetic DNA plasmid (pBARF1). We observed that immunization with pBARF1 induced both CD4$^+$ and CD8$^+$ T cell responses in both C57BL/6 and BALB/c mice. Potent serological responses were induced irrespective of animal strain. As there is no simple model to study immune responses targeting EBV$^+$ tumors in mice, we next established two BARF1$^+$ carcinoma models to allow for immune impact studies in both C57BL/6 and BALB/c mice. Using these models, we observed that immunization of pBARF1 significantly improved animal survival in the therapeutic setting. In a prechallenge immunization model, immunotherapy with pBARF1 was able to completely limit tumor growth. We demonstrated that this tumor impact was associated with the induction of CD8$^+$ T cell immunity. Finally, using an *in vivo* imaging system (IVIS), we observed that pBARF1-induced immunity cleared tumor cells as early as 2 days post-challenge. These data suggest that BARF1 may be important as a possible therapeutic target for EBV immune therapy and that its further study in this context is warranted.

RESULTS

Design and *in vitro* expression of pBARF1

Native BARF1 protein consists of 221 amino acids (Figure 1A). It contains an N-glycosylation on asparagine 95 (Asn95), which is important for protein folding and secretion and an O-glycosylation on threonine 169 (Thr169). After cleavage of the signal peptide (1–20 residue), sBARF1 (21–221 residue) is secreted as a hexamer that is complexed by three dimers in two layers.[15] The sBARF1 was shown to interfere with macrophage differentiation through its binding directly to M-CSF. Here, we studied the native *BARF1* gene, which is 100% conserved among EBV strains B95.8, GD1, and AG876, in the pBARF1 plasmid design. We synthesized the DNA plasmid by replacing the BARF1 native signal peptide sequence with an immunoglobulin E (IgE) leader sequence for enhanced expression.[26,27] The DNA sequence was codon- and RNA-optimized and cloned into a pVax expression vector (Figure 1B). After the development of the pBARF1 plasmid, we transfected the construct into 293T cells to confirm its expression *in vitro*. We observed the BARF1 protein primarily in the cell lysate, and the double bands suggested BARF1 detection both pre- and post-cleavage of the IgE signal peptide (Figure 1C).

pBARF1 elicits high titers of antigen-specific antibody responses

To determine the immunogenicity of the synthetic pBARF1, we immunized both C57BL/6 and BALB/c mice with 25 μg of pBARF1 or pVax control three times at 2-week intervals (Figure 2A). Since BARF1 is expressed on the cell surface and is mostly secreted,[28] we examined the antibody response induced by pBARF1. We collected mouse sera 1 week after each immunization and performed ELISA to measure the binding of sera to the recombinant BARF1 protein (Figures 2B and 2C). We observed that pBARF1 elicited a rapid seroconversion on day 21, 1 week after the second immunization. BARF1-binding trended higher in C57BL/6 mice than in BALB/c mice. However, the endpoint titers reached more than 1×10^5 in both strains of mice after three immunizations, with no statistical difference observed (Figure 2D).

pBARF1 induces potent antigen-specific and polyfunctional T cell responses

To evaluate the T cell response generated by pBARF1, we harvested the mice splenocytes following their final immunization (Figure 2A). Splenocytes were stimulated with BARF1 native peptides and evaluated by IFN-γ ELISpot assay. We observed significant activation of BARF1-specific IFN-γ T cell responses in the pBARF1 group, with over 1,000 spot forming units (SFU) in both C57BL/6 and BALB/c mice (Figures 3A and 3B). We analyzed the intracellular cytokine production of stimulated splenocytes by flow cytometry to study T cell phenotypes (Figures 3C–3J). We utilized Boolean gating to examine polyfunctional T cell populations that produce different cytokine combinations. We observed significant increases of IFN-γ^+, IFN-γ^+

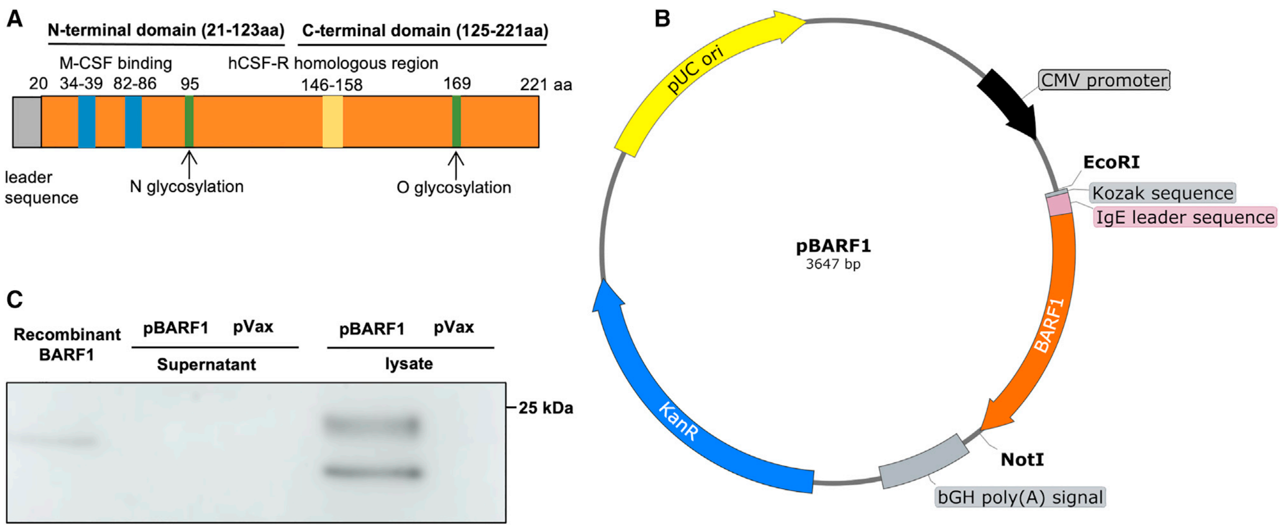

Figure 1. Design and *in vitro* expression of pBARF1

(A) Schematic representation of native BARF1 protein. (B) Depiction of pBARF1 plasmid. Kozak sequence, IgE leader sequence, and cloning sites of pBARF1 plasmid are indicated. (C) Western blot of BARF1 expression in supernatant and lysate of pBARF1-transfected 293T cells. Recombinant BARF1 protein and pVax-transfected 293T cells were used as the positive and negative controls, respectively.

tumor necrosis factor (TNF)-α^+, and IFN-γ^+ TNF-α^+ interleukin (IL)-2^+ populations in $CD8^+$ T cells from the pBARF1-immunized animals in both C57BL/6 mice and BALB/c mice, accounting for more than 1.8% of total $CD8^+$ T cells. IFN-γ^+ and IFN-γ^+ TNF-α^+ populations were activated similarly in $CD8^+$ T cells in C57BL/6 mice, while the IFN-γ^+ TNF-α^+ population was more pronounced in BALB/c mice (Figures 3C–3F). $CD4^+$ T cells were activated in the pBARF1 group but to a lesser extent than $CD8^+$ T cells (Figures 3G–3J). IFN-γ^+ TNF-α^+, IFN-γ^+ IL-2^+, TNF-α^+ IL-2^+, and IFN-γ^+ TNF-α^+ IL-2^+ populations of $CD4^+$ T cells were significantly elevated in both C57BL/6 and BALB/c mice. These results demonstrate that pBARF1 induces potent antigen-specific $CD4^+$ and $CD8^+$ T cell responses in both C57BL/6 and BALB/c mice.

pBARF1 improves mice survival in BARF1$^+$ carcinoma models

We sought to evaluate the impact of the immunity generated by immunization with pBARF1 in murine tumor models. However, there are no EBV$^+$ mouse cancer cell lines available for challenge studies as EBV does not infect mice. Therefore, we generated two tumor models for these studies by stably expressing BARF1 in carcinoma cell lines (Figure S1A). We used a retroviral vector (pBMN-I-GFP) into which we cloned the *BARF1* open reading frame (ORF) and which also expresses GFP, allowing us to follow transduction. The newly synthesized vector (pBMN-I-BARF1-GFP) was used to generate a retrovirus for transducing BARF1-GFP into MC38 and CT26 cells, which are murine colon adenocarcinoma cells syngenetic for C57BL/6 and BALB/c mice, respectively. We next performed single-cell cloning on transduced cell lines to isolate stable clonal populations for a detailed study. Using flow cytometry, we confirmed that we had a single GFP$^+$ population of CT26-BARF1 or MC38-BARF1 cells as validation of the clonality of the created cell lines (Figures S1B and S1C). To confirm *BARF1* expression levels in transduced mouse cell lines and compare the levels of expression observed with human EBV$^+$ cancer cells, we quantified *BARF1* mRNA levels by qRT-PCR. A panel of EBV-positive and -negative human cell lines were also studied side by side in this assay, including C666-1 (NPC; EBV$^+$), SUN-719 (gastric tubular adenocarcinoma; EBV$^+$), and a control AGS (gastric adenocarcinoma; EBV$^-$). *BARF1* transcripts in CT26-BARF1 and MC38-BARF1 cells (Figure S1D), as well as C666-1 and SUN-719 cells (Figure S1E), were detected at around 20 threshold cycles (Ct), suggesting a positive expression. The negative controls, CT26, MC38, and AGS cells, all showed an amplification of *BARF1* above 30 Ct, indicating background or nonspecific levels of *BARF1* expression. These data confirmed the expression of *BARF1* in the mouse cell lines following transduction and showed that the levels of expression are within the range of human EBV$^+$ cancer cells, allowing us to study the aspects of immune potency targeting these model cells.

With these novel BARF1$^+$ carcinoma models, we next studied the therapeutic efficacy of pBARF1. We injected 5×10^5 MC38-BARF1 cells subcutaneously on the flank of C57BL/6 mice and subsequently immunized them with pBARF1 or pVax on day 4 and every 2 weeks following tumor challenge (Figure 4A). We followed tumor growth over time and observed a significant decrease of tumor burden in the pBARF1-immunized mice compared with in the pVax immunized animals (Figure 4B). At the end of the study, the survival of pBARF1-treated animals was significantly improved over the control animals (Figure 4C), with 3 out of 10 animals in the pBARF1 group appearing to completely control cancer for 100 days. Additionally, we observed a similar therapeutic efficacy of pBARF1 in BALB/c mice challenged with the CT26-BARF1 cells but without complete tumor control (Figure S2). While the further characterization of these

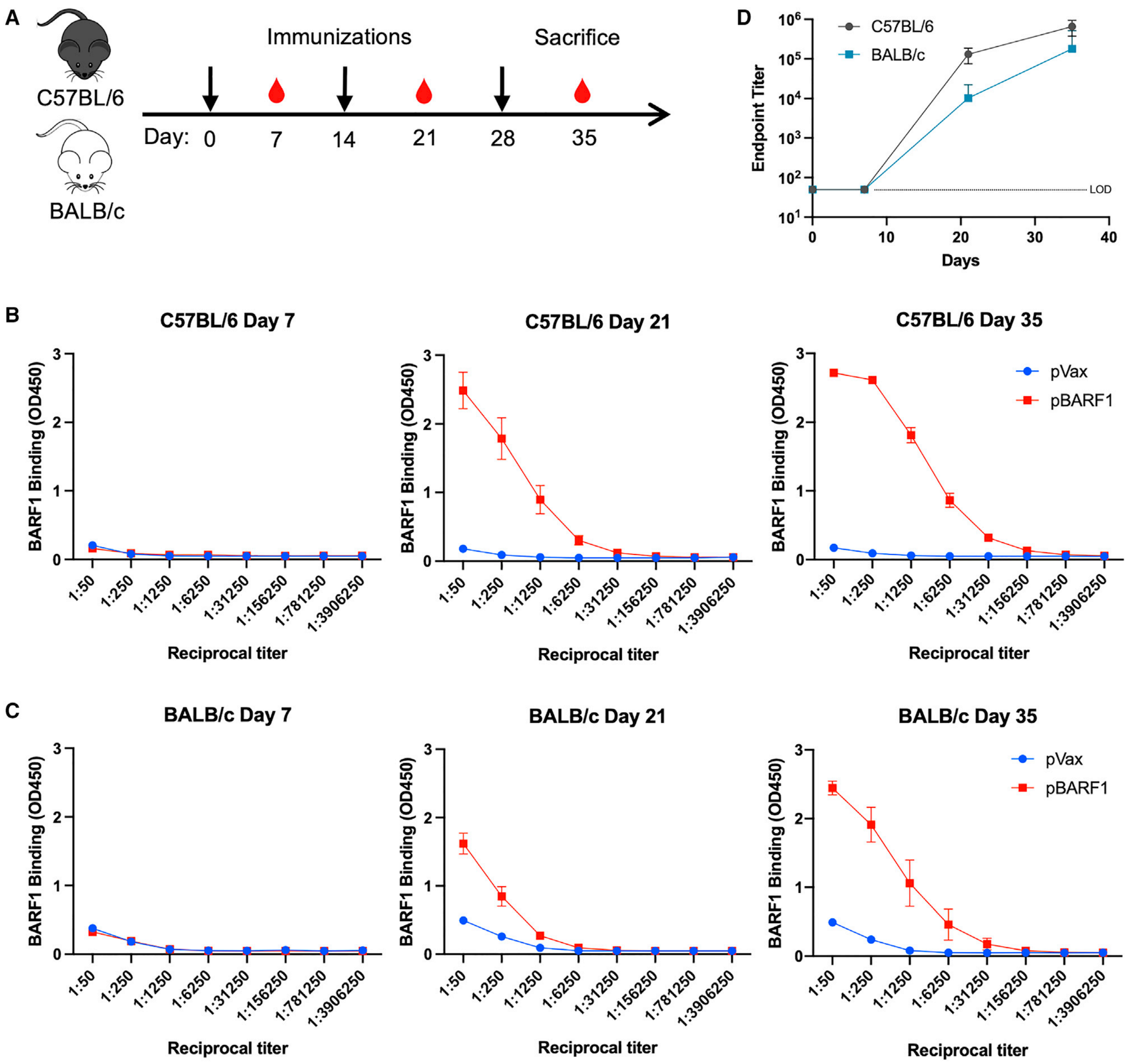

Figure 2. pBARF1 elicits high titers of antigen-specific antibody responses

(A) Outline of the immunogenicity study of pBARF1 in C57BL/6 and BALB/c mice. Sera were collected 1 week after each immunization, and splenocytes were harvested 1 week after the final immunization. (B and C) Binding of sera from immunized C57BL/6 (B) and BALB/c (C) mice to recombinant BARF1 protein over time and detected by ELISA assay. (D) Endpoint titer of mice sera determined by BARF1-binding activity from (B) and (C). n = 5 mice/group. Results are representative of two independent experiments. Error bars indicate mean ± SEM.

models is important, these data in both suggest immune responses to BARF1 can impact tumor growth of two different colon carcinoma lines in two different genetic backgrounds that both express BARF1.

Single immunization of pBARF1 completely suppresses tumor growth in a CD8$^+$ T cell-dependent manner

Next, we evaluated the efficacy of pBARF1 in a prechallenge immunization model. We immunized BALB/c mice with one, two, or three doses of pBARF1 or pVax 1 week before tumor inoculation (Figure 5A). To explore the contribution of different components of the immune response to tumor rejection, we also depleted CD4$^+$ or CD8$^+$ T cells starting 1 day before tumor challenge in additional pBARF1 immunized groups. We observed that mice receiving one, two, or three doses of pBARF1 completely suppressed tumor growth, while the pVax group exhibited significantly higher tumor volume (Figure 5B). CD4$^+$ T cell depletion in mice immunized with pBARF1

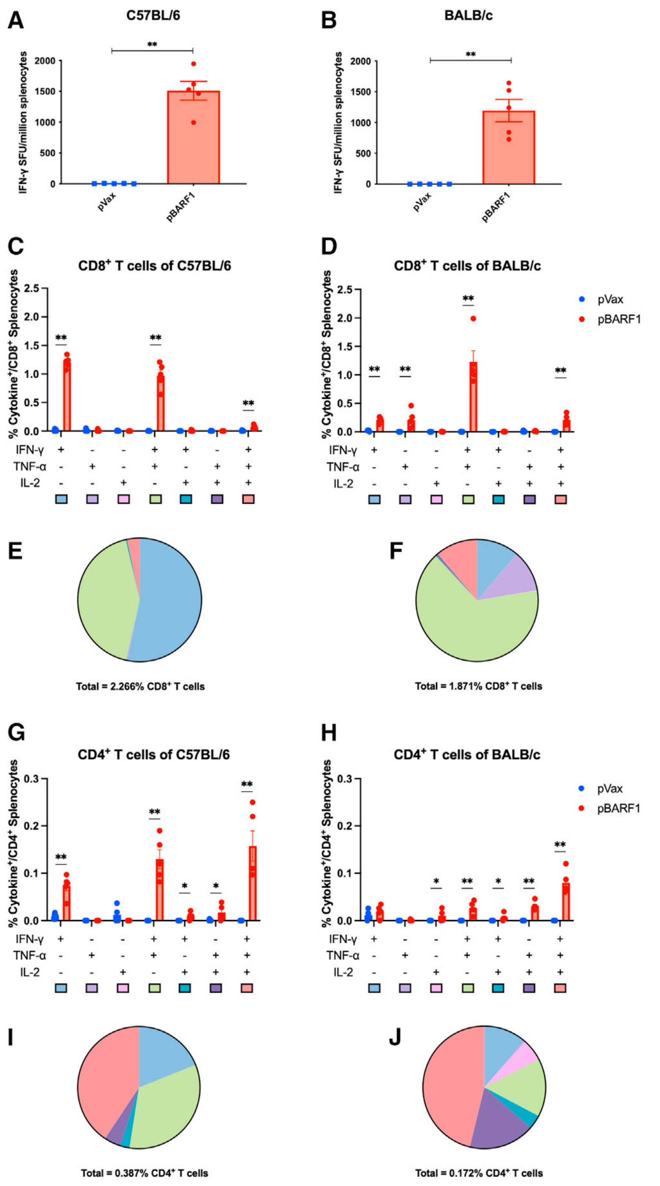

Figure 3. pBARF1 induces potent antigen-specific polyfunctional T cell responses

(A–J) Splenocytes from mice immunized with pBARF1 or pVax (outlined in Figure 2A) were stimulated by native BARF1 peptides. (A and B) IFN-γ ELISpot assay of stimulated splenocytes in C57BL/6 and BALB/c mice. (C, D, G, and H) Intracellular staining of polyfunctional $CD8^+$ and $CD4^+$ T cells producing different cytokine combinations. (E, F, I, and J) Pie charts represent the proportions of each cytokine-producing T cell population of pBARF1 immunized mice in (C), (D), (G), and (H). Percentage of total polyfunctional T cell populations in $CD4^+$ or $CD8^+$ T cells are indicated. Significance was determined using the nonparametric Mann-Whitney U test. $*p < 0.05$, $**p < 0.01$. n = 5 mice/group. Results are representative of two independent experiments. Error bars indicate mean ± SEM.

did not affect tumor control. However, mice with $CD8^+$ T cell depletion completely lost tumor control and exhibited a high tumor burden similar to the controls (Figure 5B), suggesting that $CD8^+$ T cells are important for the therapeutic efficacy of pBARF1. Immunization one, two, or three times with pBARF1 resulted in 100% survival rates following the challenge (Figure 5C). Furthermore, to evaluate the memory immune responses, the mice that received one, two, or three doses of pBARF1 and survived tumor challenge were randomized and rechallenged with either CT26 or CT26-BARF1 cells on day 446 post-initial challenge (Figures 5A and 5C). We observed that the mice rejected CT26-BARF1 but not native CT26 cells, indicating that long-term anti-BARF1 immunity was specifically induced (Figure 5D). Additionally, protection after a single immunization with pBARF1 was observed when studied in the MC38-BARF1 model (Figure S3).

Finally, to study tumor clearance over time, we immunized animals and then monitored tumor growth *in vivo* by using the IVIS. For this study, we transduced CT26-BARF1 or CT26 cells with cytomegalovirus (CMV)-Firefly luciferase lentivirus and developed CT26-BARF1-Luc or CT26-Luc cell lines for challenge studies. We immunized BALB/c mice with three doses of either pBARF1 or pVax control plasmid and challenged both groups with the CT26-BARF1-luciferase (Luc) or CT26-Luc cells (Figure 6A). Mice immunized with pBARF1 showed clearance of CT26-BARF1-Luc as early as 2 days post-challenge (Figure 6B). By day 10, all animals exhibited no detectable tumor burden, and tumor-free survival was observed (Figures 6C and 6D). In contrast, we observed rapid tumor growth in the pVax group. The second control group, in which mice were immunized with pBARF1 and challenged with CT26-Luc, also showed cancer progression (Figures 6B–6D). Together, these data indicate that pBARF1-mediated T cell immunity was focused on the BARF1 displayed on the tumor cell and not an irrelevant cell target, supporting the specificity of the anti-tumor response.

DISCUSSION

Here, we provide the first study on the immune impact of BARF1 in a mouse immunotherapy model. We observed that immunization with a DNA vaccine encoding BARF1 can drive antigen-specific immunity against BARF1 and impact cancer progression in a model system *in vivo*. The impact of the challenge appears to be primarily dependent on the $CD8^+$ T cell response, which was associated with both short- and long-term protection. Using a prechallenge immunization model, we showed that one dose of pBARF1 induced complete control of cancer progression. These results provide supportive evidence that immunity to BARF1 may be capable of targeting EBV cells that express BARF1 for immune clearance, which has implications for immune therapy of EBV-driven cancers.

Structural studies have shown that BARF1 can form as a hexamer, which acts as a decoy receptor for human M-CSF.[15] BARF1 interferes with M-CSF and receptor binding, and this interaction disturbs monocyte differentiation, which potentially affects macrophage polarization in the tumor microenvironment (TME). However, there is limited knowledge regarding how BARF1 affects the TME status in NPC or EBVaGC. Here, we observed potent humoral responses induced by pBARF1, suggesting strong immune reactivity (Figure 2).

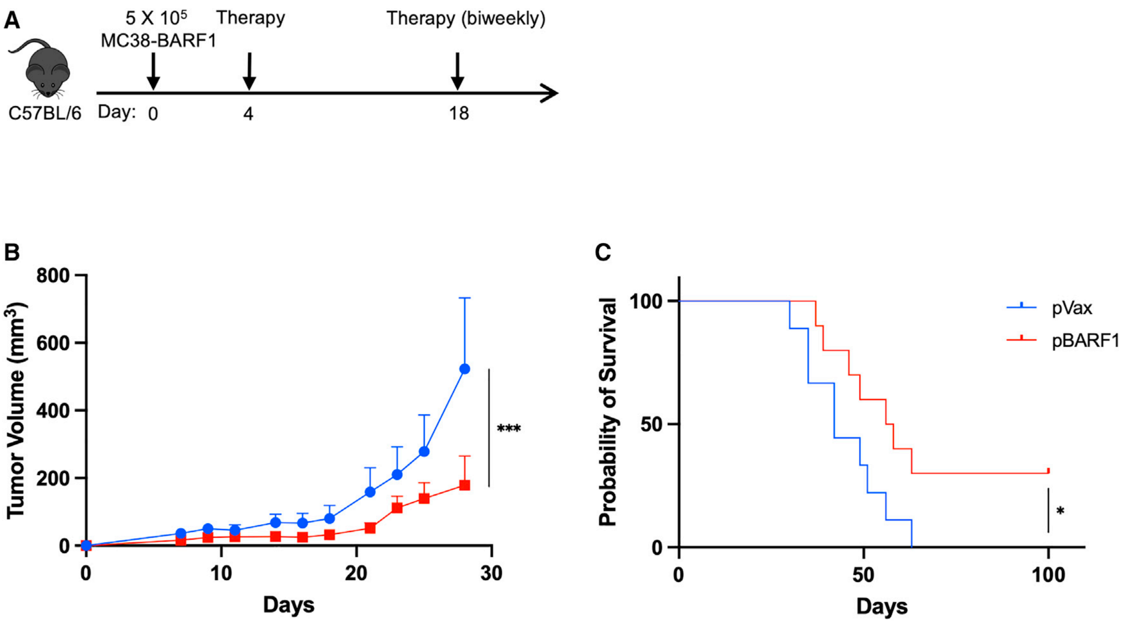

Figure 4. Immunization with pBARF1 improves survival in the therapeutic tumor model in C57BL/6 mice

(A) Study outline for the therapeutic tumor model. The mice were injected with MC38-BARF1 cells and immunized with pBARF1 biweekly starting on day 4. (B) Tumor volume measurements over time for mouse study described in (A). (C) Survival curve for the mouse study described in (A). Significance for tumor volume was determined by two-way ANOVA. Significance for survival was determined by the log rank test. *$p < 0.05$, ***$p < 0.001$. n = 9–10 mice/group. Error bars indicate mean ± SEM.

However, antibody responses were not sufficient to protect mice from tumor challenge, as $CD8^+$ T cell depletion completely reversed the therapeutic effects of pBARF1 (Figures 5A–5C). As BARF1 was reported not to interfere with differentiation of mouse monocytes,[15] the potential therapeutic effect of humoral responses against BARF1 and its impact on the TME need to be further investigated in a more relevant model that can assess the impact of the serology induced on M-CSF-BARF1 interactions. The development of such a model is important.

Many studies describe the importance of CTL responses in the context of cancer immunotherapy.[29] In regard to immune therapy with DNA, we previously reported on a phase IIb study testing VGX3100, a human papillomavirus (HPV) DNA vaccine immune therapy, for women with high-grade cervical dysplasia. We showed that immunization with VGX-3100 induced potent HPV 16 and 18, E6 and E7-specific CTL responses, which are the antigens encoded in the vaccine.[30] Functional T cell responses were also identified as important biomarkers for patient response.[31,32] Similarly, we observed that DNA immunotherapy (MEDI0457) induced tumor-infiltrating T cells in patients with HPV-associated advanced head and neck squamous cell cancer (HNSCCa), highlighting the ability of this approach to drive virally relevant CTLs as tools for immune therapy of a virally driven cancer.[33] Consistent with these studies, here, we found that pBARF1 induced potent CTL responses in two strains of mice and that these T cells responses were correlated with clearance of MC38-BARF1 or CT26-BARF1 tumors (Figure 3, Figure 5, and Figure S3). These data further highlight the importance of $CD8^+$ T cell effector function in tumor control (Figure 5B-5C). We observed potentially increased tumor control in C57BL/6 mice (Figure 4), which had a $CD8^+$ T cell profile dominated by an IFN-γ^+ population compared with BALB/c mice, which exhibited slightly decreased tumor control (Figure S2), and lower total $CD8^+$ T cell IFN-γ responses than those observed in the C57BL/6 model (Figures 3C–3F).

This therapeutic outcome could be of relevance to human immunotherapy, as the examination of some EBV-infected patients showed that BARF1 induced both $CD4^+$ and $CD8^+$ T cell responses as evidenced in EBV seropositive patients.[19,34] Infection-induced BARF1-specific CTLs from some patients were shown to kill EBV and cancer *in vitro*. However, these studies did not test if the CTLs induced could influence tumor growth *in vivo* or study their induction. Our studies begin to address these questions. Previously, for HPV immune therapy, there was a similar model issue for immunotherapy studies, as HPV does not infect murine cell lines. TC-1, a mouse cancer cell line, was developed by transfection to carry cDNA-encoding E6 and E7 proteins of HPV16.[35] This cell line allows for studies of immunotherapy in mice with competent immune systems in the context of a growing tumor.[36] Using the TC-1 model, immunotherapies targeting HPV were advanced, some of which have later shown promising efficacy in clinical studies against HPV-associated diseases, supporting the translational relevance of this model.[30,33,37] Therefore, with a similar principle, we generated the $BARF1^+$ tumor models as surrogates for human tumors expressing BARF1 with caveats that these models are matched to mouse major histocompatibility complexes (MHCs), not human MHCs. As a first pass here, we observed that

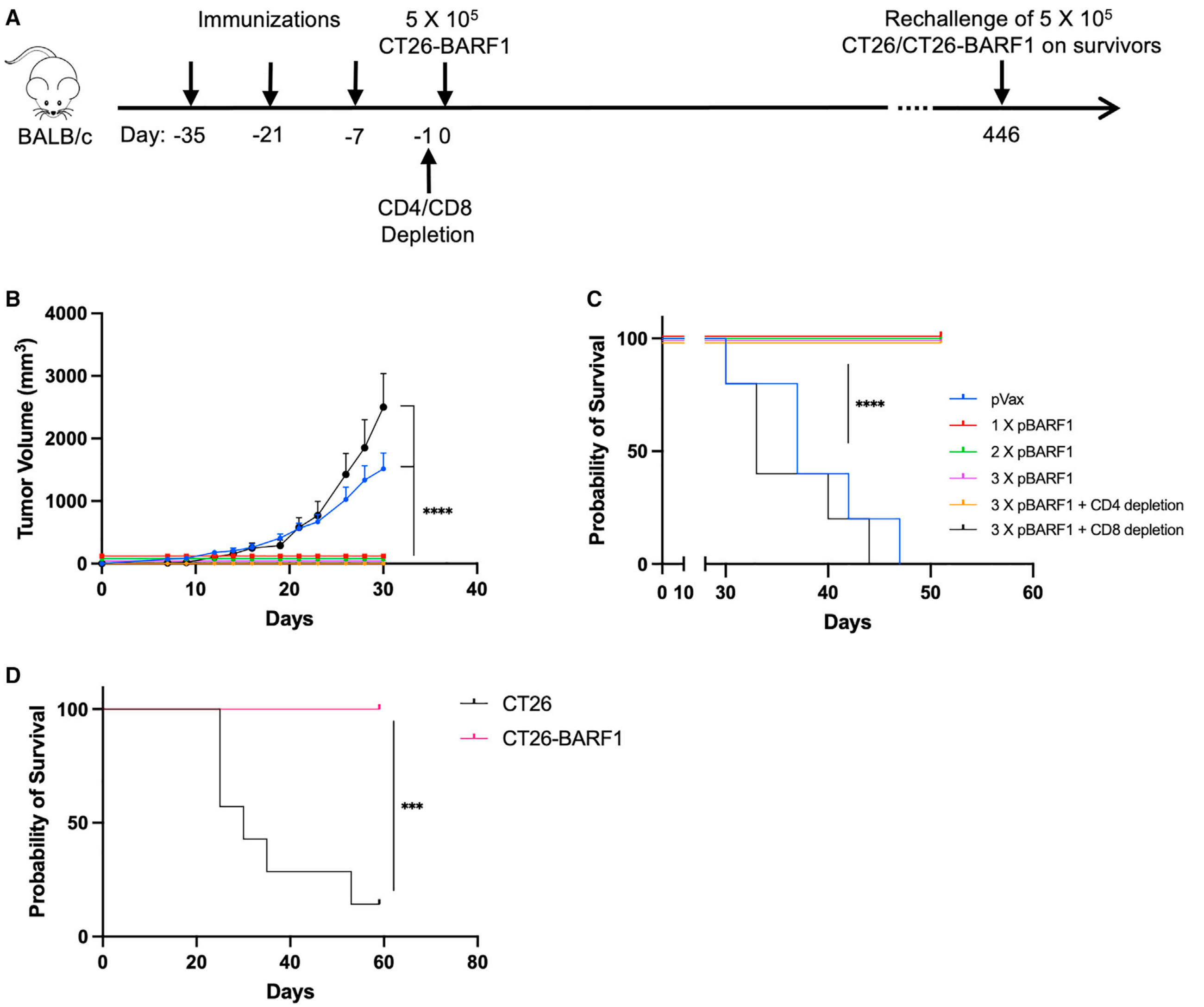

Figure 5. pBARF1 completely suppresses cancer progression through CD8$^+$ T cells and maintains long-term tumor control

(A) Study outline for the prechallenge immunization model. The mice were immunized with one, two, or three doses of pBARF1 2 weeks apart. CT26-BARF1 cells were injected 1 week after the final immunization, and anti-CD4 or anti-CD8 antibodies were given 1 day before the tumor challenge. (B and C) Tumor volume measurements (B) and survival plot (C) of the initial challenge study described in (A). n = 5 mice/group. Results are representative of two independent experiments. (D) Mice received one, two, or three doses of pBARF1, and those who survived tumor challenge in (C) were randomized and rechallenged with CT26 or CT26-BARF1 cells on day 446 after the initial tumor challenge (A). The survival curve is shown. n = 7–8 mice/group. Significance for tumor volume was determined by two-way ANOVA. Significance for survival was determined by the log rank test. ***$p < 0.001$, ****$p < 0.0001$. Error bars indicate mean ± SEM.

the transduced and cloned mouse carcinoma cell lines developed, CT26-BARF1 and MC38-BARF1, expressed similar levels of *BARF1* compared with human NPC and EBVaGC cell lines, C666-1 and SUN719, which have been previously obtained from EBV$^+$ cancer patients (Figures S1D and S1E). While more development is important, this data supports, in part, aspects of physiological similarity between our models and human EBV$^+$ cancer.

DNA antigen immunogenicity has been enhanced by various strategies.[38] Here, we have adopted codon and RNA optimization, Kozak sequence, IgE leader sequence, and adaptive electroporation delivery for the pBARF1 antigen to enhance expression and immunogenicity.[39,40] Other strategies to enhance DNA immunotherapy include formulation with adjuvants, such as IL-12, and nanoparticle assembly of the antigen, among others, as recently reported.[27,41,42] As the N-terminal domain of BARF1 can activate anti-apoptotic *Bcl-2* expression and bind to M-CSF for immune modulation, domains or fragments of BARF1 that contain immune dominant B cell or T cell epitopes can be investigated for future studies.[14] For immunotherapy in humans, additional important

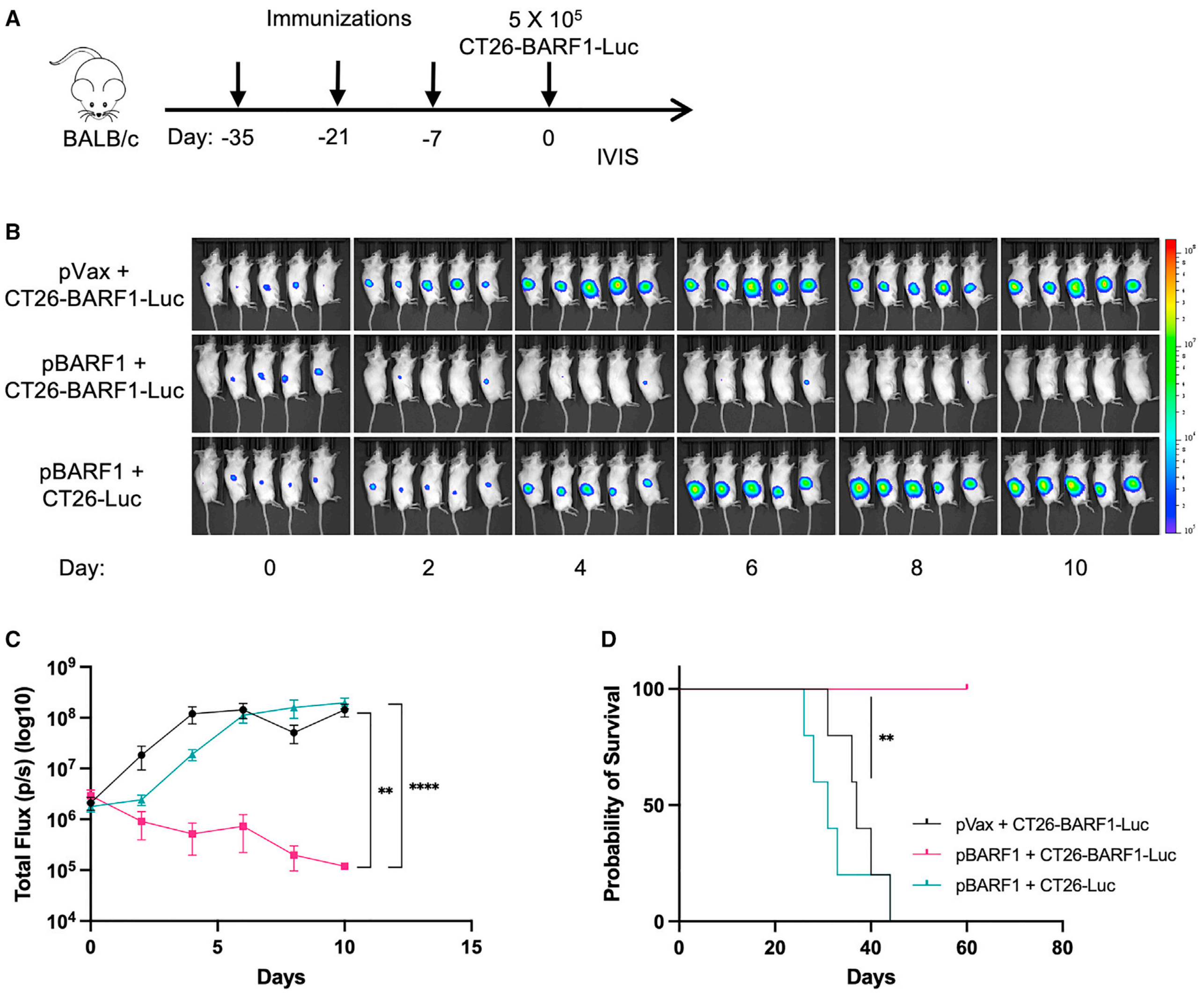

Figure 6. pBARF1-induced immunity mediates rapid clearance of cancer cells

(A) Study outline for the prechallenge immunization model with IVIS. The mice were immunized with three doses of pBARF1 2 weeks apart. CT26-Luc or CT26-BARF1-Luc cells were injected 1 week after the final immunization. (B and C) IVIS imaging of tumor-bearing mouse (B) and quantification of the bioluminescence signal (C) captured in (B). (D) Survival curve for the mouse study described in (A). Significance for total flux was determined by two-way ANOVA. Significance for survival was determined by the log rank test. $^{**}p < 0.01$, $^{****}p < 0.0001$. n = 5 mice/group. Error bars indicate mean ± SEM.

antigens as part of an immune cocktail may be important. Combining BARF1 with other EBV-latent proteins, such as EBNA1, LMP1, and LMP2, could be considered for immunotherapy studies.[9,43,44] This multi-target approach would cover EBV^+ cancer cells at different latency phases with diverse protein expression levels, thus possibly providing an additional advantage for limiting tumor escape.

Combined immunotherapy has been investigated in both preclinical and clinical studies. Pembrolizumab, a checkpoint inhibitor against PD1, was approved for recurrent and metastatic NPC, but the overall response rate is only 26.3%.[10] The non-responders are likely to be patients with low tumor-infiltrating lymphocytes (TILs). Although the immunosuppressive TME can be reshaped by anti-PD1, the CTLs might not be abundant enough to control cancer cells. Combining pBARF1 with checkpoint inhibitors or other T cell-related immunotherapies may support TIL abundance and enhance tumor clearance synergistically.[45–48]

In conclusion, we provide evidence in a mouse model of the relevance of BARF1 in immunotherapy for EBV-driven cancer. The immune potency of these vaccinations was highly impactful. Further study of BARF1 and immunotherapy for EBV is important and may represent a new tool to expand treatment options for patients with EBV-associated cancer.

MATERIALS AND METHODS

DNA plasmids

The pBARF1 plasmid construct was designed by adding a Kozak sequence and an IgE leader sequence to the N terminus of the native BARF1 protein sequence (amino acid 21–221; Uniprot: P03228). It was codon- and RNA-optimized and cloned into the modified pVax vector between restriction site EcoRI and NotI (GenScript, Piscataway, NJ, USA). For the pBMN-I-BARF1-GFP plasmid, the native BARF1 sequence was codon- and RNA-optimized and inserted (GenScript) into a retroviral vector, pBMN-I-GFP (Nolan Lab; Addgene plasmid #1736, Watertown, MA, USA). The plasmid map of pBARF1 was generated by SnapGene v.5.3.2 (San Diego, CA, USA).

Cell lines, transfection, and transduction

CT26, MC38, HEK293T, Phoenix, and AGS cells were obtained from American Type Culture Collection (ATCC, Manassas, VA, USA). SNU-719 was purchased from Korean Cell Line Bank (Seoul, South Korea). C666-1 was originally provided by G. Tsao's lab at Hong Kong University (Hong Kong, China). For *in vitro* transfection, Lipofectamine 3000 (Thermo Fisher Scientific, Waltham, MA, USA) was used following the manufacturer's instructions. For transduction of BARF1 into CT26 and MC38, the retrovirus was first produced in Phoenix cells transfected with pBMN-I-BARF1-GFP plasmid and then added to CT26 and MC38 cells. Single-cell cloning by limiting dilution was used to select GFP^+ clones of transduced CT26 and MC38 cells. For transduction of luciferase into CT26-BARF1 and MC38-BARF1 cells, a CMV-Firefly Luc lentivirus (Cellomics Technology, Halethorpe, MD, USA) was used following the manufacturer's instructions. All cell lines were maintained in RPMI 1640 with 10% fetal bovine serum (FBS) and 1% penicillin and streptomycin (R10). They were routinely tested for *Mycoplasma* contamination.

Immunoblotting

Recombinant BARF1 protein was synthesized by GenScript. Cell lysis, protein extraction, denaturation, and western blotting were done as previously described.[27] Polyvinylidene fluoride (PVDF) membranes were blotted with mouse anti-BARF1 serum as the primary antibody and goat anti-mouse IgG-horseradish peroxidase (HRP; ab6789, Abcam, Cambridge, UK) as the secondary antibody. The signal was developed by SignalFire ECL reagent (Cell Signaling Technology, Danvers, MA, USA), and images were captured by Amersham Imager 680 (GE Healthcare Life Sciences, Piscataway, NJ, USA).

Reverse transcription and quantitative PCR

Total RNA was isolated from cells by RNeasy Mini Kit (Qiagen, Hilden, Germany), and cDNA was synthesized using high-Capacity cDNA Reverse Transcription Kit (Thermo Fisher Scientific), following the manufacturer's instructions. The mRNA expression of *BARF1* was determined by quantitative PCR using the Power SYBR Green Master Mix (Thermo Fisher Scientific) and QuantStudio 5 PCR System (Thermo Fisher Scientific). Primers were synthesized by Integrated DNA Technologies (Coralville, IA, USA): transduced *BARF1*, 5′-CTTCATCGAGTGGCCCTTT-3′ (forward) and 5′-CTTCATCCTGCACAGGTAGTT-3′ (reverse); native *BARF1* 5′-GCCTCTAACGCTGTCTGTCC-3′ (forward) and 5′-GAGAGGCTCCCATCCTTTTC-3′ (reverse).[49]

Animal immunization

C57BL/6 and BALB/c mice were purchased from The Jackson Laboratory (Bar Harbor, ME, USA). Twenty-five μg of DNA plasmid (pBARF1 or pVax) in 30 μL water was injected into the tibialis anterior (TA) muscle followed by delivery of two 0.1 Amp electric constant current square-wave pulses by the CELECTRA-3P device (Inovio Pharmaceuticals, Plymouth Meeting, PA, USA). The immunization schedule is indicated in each figure. All procedures were done under the guidelines of the Wistar Institute Animal Care and Use Committee (Philadelphia, PA, USA).

Tumor studies

C57BL/6 and BALB/c mice were immunized as described in the previous section at multiple doses before or after tumor challenge, as illustrated in each figure. 5×10^5 of CT26, CT26-BARF1, MC38, MC38-BARF1, CT26-Luc, or CT26-BARF1-Luc cells (all under five passages) were injected subcutaneously into the right flank of the animals. Tumors were measured three times a week by electric calipers, and tumor volume was calculated by the formula: $\text{volume} = 0.5 \times \text{height} \times \text{width}^2$. Mice were euthanized when any dimension of the tumor reached 20 mm. For depletion of $CD4^+$ and $CD8^+$ T cell, 200 μg of anti-CD8a (YTS169.4, BioXCell, Lebanon, NH, USA) and anti-CD4 (GK1.5, BioXCell) antibodies were injected intraperitoneally to each mouse twice a week until the end of the study. For the IVIS study, 200 μL of D-Luciferin (GoldBio, St. Louis, MO, USA) was injected intraperitoneally into each mouse, and bioluminescence signal was captured by IVIS SpectrumCT (PerkinElmer, Waltham, MA, USA). IVIS images and bioluminescence signals were analyzed by Living Image v4.7.3 (PerkinElmer).

ELISpot assay

Spleens from immunized mice were harvested and dissociated by a stomacher. Red blood cells were removed by ACK lysing buffer (Thermo Fisher Scientific). The splenocytes were filtered and counted. 2×10^5 splenocytes were plated into each well on Mouse IFN-γ ELISpot^{PLUS} plates (Mabtech, Stockholm, Sweden) and stimulated for 20 h with a pool of 33 BARF1 peptides (15 mer peptides overlapping by 9 amino acids covering the full-length native BARF1 protein, GenScript). Cells were stimulated with 5 μg/mL of each peptide in complete media (R10). The spots were developed according to the manufacturer's instructions. R10 and cell stimulation cocktails (Thermo Fisher Scientific) were used for negative and positive controls, respectively. Spots were scanned and quantified by ImmunoSpot Macro Analyzer (Cellular Technology Limited, Cleveland, OH, USA). SFU per million cells was calculated by subtracting the negative control wells.

Intracellular cytokine staining and flow cytometry

Splenocytes were stimulated by BARF1 peptides for 5 h with a protein transport inhibitor (Thermo Fisher Scientific). A cell stimulation cocktail and R10, with a protein transport inhibitor, were used as positive and negative controls, respectively. After stimulation, cells were stained with LIVE/DEAD violet (Thermo Fisher Scientific) for viability. CD3e (17A2), CD4 (RM4-5), CD8b (YTS156.7.7), IFN-γ (XMG1.2), TNF-α (MP6-XT22), and IL-2 (JES56-5H4) fluorochrome-conjugated antibodies (all from BioLegend, San Diego, CA, USA) were used for surface and intracellular staining. The samples were run on an 18-color LSRII flow cytometer (BD Biosciences, Franklin Lakes, NJ, USA) and analyzed by FlowJo v.10.8.1 (BD Biosciences).

Binding ELISA

NUNC MaxiSorp 96-well plates (Thermo Fisher Scientific) were coated with 1 μg/mL recombinant BARF1 protein (GenScript) in PBS overnight at 4°C. The plates were washed with PBS-0.5% Tween 20 and blocked with PBS 10% FBS. Next, the plates were incubated with diluted mouse sera for 2 h and with goat anti-mouse IgG-HRP (Abcam) for 1 hour at room temperature. TMB substrate (Thermo Fisher Scientific) was used to develop the binding signal.

Statistical analysis

All statistics were analyzed using Prism v.9.3.0 (GraphPad, San Diego, CA, USA). Error bars represent mean ± SEM. For differences between the means of groups, significance was determined by the nonparametric Mann-Whitney U test. For mouse tumor volume measurements, significance was determined by two-way ANOVA. For mouse survival studies, significance was determined by the log rank test.

SUPPLEMENTAL INFORMATION

Supplemental information can be found online at https://doi.org/10.1016/j.omto.2021.12.017.

ACKNOWLEDGMENTS

We are grateful to the Wistar Institute's Animal Facility, Flow Cytometry Facility, and Imaging Facility for providing technical support. Funding support for the Wistar Institute core facilities was provided by Cancer Center Support Grant P30 CA010815. This work was supported by the Wistar/UPenn SPORE under the Skin Cancer Developmental Research Project grant (P50 CA174523 to D.B.W.), the W.W. Smith Charitable Trust Professorship in Cancer Research (to D.B.W.), and a grant from INOVIO Pharmaceuticals (to D.B.W.). The graphical abstract and some figures were created with BioRender.com.

AUTHOR CONTRIBUTIONS

X.Z., A.P.-P., and D.B.W. conceived the study. X.Z. and A.P.-P. designed the experiments. X.Z., A.P.-P., Z.X., E.N.G., and A.P. developed the methodology. P.M.L. provided resources and reagents. X.Z., A.P.-P., K.Z., Z.X., K.Y., P.B., D.B., D.H.P., K.L., and M.A.B performed the experiments. X.Z. and D.B.W. wrote the manuscript, and X.Z., A.P.-P., Z.X., P.B., D.B., E.N.G., and D.B.W. reviewed and edited the manuscript. D.B.W. provided supervision.

DECLARATION OF INTEREST

D.B.W. has received grant funding, participates in industry collaborations, has received speaking honoraria, and has received fees for consulting, including serving on scientific review committees and board series. Remuneration received by D.B.W. includes direct payments, stock, or stock options, and in the interest of disclosure, D.B.W. discloses the following paid associations with commercial partners: Geneos (advisory board), Astrazeneca (advisory board, speaker), Inovio (BOD, SRA, Stock), Sanofi (advisory board), and BBI (advisory board). All other authors declare no competing interests.

REFERENCES

1. de Martel, C., Georges, D., Bray, F., Ferlay, J., and Clifford, G.M. (2020). Global burden of cancer attributable to infections in 2018: a worldwide incidence analysis. Lancet Glob. Health *8*, e180–e190.
2. Young, L.S., Yap, L.F., and Murray, P.G. (2016). Epstein-Barr virus: more than 50 years old and still providing surprises. Nat. Rev. Cancer *16*, 789–802.
3. Shannon-Lowe, C., and Rickinson, A. (2019). The global landscape of EBV-associated tumors. Front. Oncol. *9*, 713.
4. Zhang, Y., Chen, L., Hu, G.Q., Zhang, N., Zhu, X.D., Yang, K.Y., Jin, F., Shi, M., Chen, Y.P., Hu, W.H., et al. (2019). Gemcitabine and cisplatin induction chemotherapy in nasopharyngeal carcinoma. N. Engl. J. Med. *381*, 1124–1135.
5. Cancer Genome Atlas Research Network (2014). Comprehensive molecular characterization of gastric adenocarcinoma. Nature *513*, 202–209.
6. Smith, C., Tsang, J., Beagley, L., Chua, D., Lee, V., Li, V., Moss, D.J., Coman, W., Chan, K.H., Nicholls, J., et al. (2012). Effective treatment of metastatic forms of Epstein-Barr virus-associated nasopharyngeal carcinoma with a novel adenovirus-based adoptive immunotherapy. Cancer Res. *72*, 1116–1125.
7. Fae, D.A., Martorelli, D., Mastorci, K., Muraro, E., Dal Col, J., Franchin, G., Barzan, L., Comaro, E., Vaccher, E., Rosato, A., et al. (2016). Broadening specificity and enhancing cytotoxicity of adoptive T cells for nasopharyngeal carcinoma immunotherapy. Cancer Immunol. Res. *4*, 431–440.
8. Turrini, R., Merlo, A., Martorelli, D., Fae, D.A., Sommaggio, R., Montagner, I.M., Barbieri, V., Marin, O., Zanovello, P., Dolcetti, R., et al. (2017). A BARF1-specific mAb as a new immunotherapeutic tool for the management of EBV-related tumors. Oncoimmunology *6*, e1304338.
9. Taylor, G.S., Jia, H., Harrington, K., Lee, L.W., Turner, J., Ladell, K., Price, D.A., Tanday, M., Matthews, J., Roberts, C., et al. (2014). A recombinant modified vaccinia ankara vaccine encoding Epstein-Barr virus (EBV) target antigens: a phase I trial in UK patients with EBV-positive cancer. Clin. Cancer Res. *20*, 5009–5022.
10. Hsu, C., Lee, S.H., Ejadi, S., Even, C., Cohen, R.B., Le Tourneau, C., Mehnert, J.M., Algazi, A., van Brummelen, E.M.J., Saraf, S., et al. (2017). Safety and antitumor activity of pembrolizumab in patients with programmed death-ligand 1-positive nasopharyngeal carcinoma: results of the KEYNOTE-028 study. J. Clin. Oncol. *35*, 4050–4056.
11. Chia, W.K., Wang, W.W., Teo, M., Tai, W.M., Lim, W.T., Tan, E.H., Leong, S.S., Sun, L., Chen, J.J., Gottschalk, S., et al. (2012). A phase II study evaluating the safety and efficacy of an adenovirus-DeltaLMP1-LMP2 transduced dendritic cell vaccine in patients with advanced metastatic nasopharyngeal carcinoma. Ann. Oncol. *23*, 997–1005.
12. Decaussin, G., Sbih-Lammali, F., de Turenne-Tessier, M., Bouguermouh, A., and Ooka, T. (2000). Expression of BARF1 gene encoded by Epstein-Barr virus in nasopharyngeal carcinoma biopsies. Cancer Res. *60*, 5584–5588.
13. zur Hausen, A., Brink, A.A., Craanen, M.E., Middeldorp, J.M., Meijer, C.J., and van den Brule, A.J. (2000). Unique transcription pattern of Epstein-Barr virus (EBV) in EBV-carrying gastric adenocarcinomas: expression of the transforming BARF1 gene. Cancer Res. *60*, 2745–2748.

14. Blanco, R., and Aguayo, F. (2020). Role of BamHI-A rightward frame 1 in Epstein-Barr virus-associated epithelial malignancies. Biology (Basel) *9*, 461.

15. Shim, A.H., Chang, R.A., Chen, X., Longnecker, R., and He, X. (2012). Multipronged attenuation of macrophage-colony stimulating factor signaling by Epstein-Barr virus BARF1. Proc. Natl. Acad. Sci. U S A *109*, 12962–12967.

16. Sall, A., Caserta, S., Jolicoeur, P., Franqueville, L., de Turenne-Tessier, M., and Ooka, T. (2004). Mitogenic activity of Epstein-Barr virus-encoded BARF1 protein. Oncogene *23*, 4938–4944.

17. Wei, M.X., de Turenne-Tessier, M., Decaussin, G., Benet, G., and Ooka, T. (1997). Establishment of a monkey kidney epithelial cell line with the BARF1 open reading frame from Epstein-Barr virus. Oncogene *14*, 3073–3081.

18. Tanner, J.E., Wei, M.X., Alfieri, C., Ahmad, A., Taylor, P., Ooka, T., and Menezes, J. (1997). Antibody and antibody-dependent cellular cytotoxicity responses against the BamHI A rightward open-reading frame-1 protein of Epstein-Barr virus (EBV) in EBV-associated disorders. J. Infect. Dis. *175*, 38–46.

19. Martorelli, D., Houali, K., Caggiari, L., Vaccher, E., Barzan, L., Franchin, G., Gloghini, A., Pavan, A., Da Ponte, A., Tedeschi, R.M., et al. (2008). Spontaneous T cell responses to Epstein-Barr virus-encoded BARF1 protein and derived peptides in patients with nasopharyngeal carcinoma: bases for improved immunotherapy. Int. J. Cancer *123*, 1100–1107.

20. Kalra, M., Gerdemann, U., Luu, J.D., Ngo, M.C., Leen, A.M., Louis, C.U., Rooney, C.M., and Gottschalk, S. (2019). Epstein-Barr virus (EBV)-derived BARF1 encodes CD4- and CD8-restricted epitopes as targets for T-cell immunotherapy. Cytotherapy *21*, 212–223.

21. Dasari, V., Sinha, D., Neller, M.A., Smith, C., and Khanna, R. (2019). Prophylactic and therapeutic strategies for Epstein-Barr virus-associated diseases: emerging strategies for clinical development. Expert Rev. Vaccines *18*, 457–474.

22. Comoli, P., Pedrazzoli, P., Maccario, R., Basso, S., Carminati, O., Labirio, M., Schiavo, R., Secondino, S., Frasson, C., Perotti, C., et al. (2005). Cell therapy of stage IV nasopharyngeal carcinoma with autologous Epstein-Barr virus-targeted cytotoxic T lymphocytes. J. Clin. Oncol. *23*, 8942–8949.

23. Lin, C.L., Lo, W.F., Lee, T.H., Ren, Y., Hwang, S.L., Cheng, Y.F., Chen, C.L., Chang, Y.S., Lee, S.P., Rickinson, A.B., et al. (2002). Immunization with Epstein-Barr virus (EBV) peptide-pulsed dendritic cells induces functional CD8+T-cell immunity and may lead to tumor regression in patients with EBV-positive nasopharyngeal carcinoma. Cancer Res. *62*, 6952–6958.

24. Si, Y., Deng, Z., Lan, G., Du, H., Wang, Y., Si, J., Wei, J., Weng, J., Qin, Y., Huang, B., et al. (2016). The safety and immunological effects of rAd5-EBV-LMP2 vaccine in nasopharyngeal carcinoma patients: a phase I clinical trial and two-year follow-up. Chem. Pharm. Bull. (Tokyo) *64*, 1118–1123.

25. Wojtak, K., Perales-Puchalt, A., and Weiner, D.B. (2019). Novel synthetic DNA immunogens targeting latent expressed antigens of Epstein-Barr virus elicit potent cellular responses and inhibit tumor growth. Vaccines (Basel) *7*, 44.

26. Perales-Puchalt, A., Wojtak, K., Duperret, E.K., Yang, X., Slager, A.M., Yan, J., Muthumani, K., Montaner, L.J., and Weiner, D.B. (2019). Engineered DNA vaccination against follicle-stimulating hormone receptor delays ovarian cancer progression in animal models. Mol. Ther. *27*, 314–325.

27. Xu, Z., Wise, M.C., Chokkalingam, N., Walker, S., Tello-Ruiz, E., Elliott, S.T.C., Perales-Puchalt, A., Xiao, P., Zhu, X., Pumroy, R.A., et al. (2020). In vivo assembly of nanoparticles achieved through synergy of structure-based protein engineering and synthetic DNA generates enhanced adaptive immunity. Adv. Sci. *7*, 2070044.

28. Houali, K., Wang, X., Shimizu, Y., Djennaoui, D., Nicholls, J., Fiorini, S., Bouguermouh, A., and Ooka, T. (2007). A new diagnostic marker for secreted Epstein-Barr virus encoded LMP1 and BARF1 oncoproteins in the serum and saliva of patients with nasopharyngeal carcinoma. Clin. Cancer Res. *13*, 4993–5000.

29. Farhood, B., Najafi, M., and Mortezaee, K. (2019). CD8(+) cytotoxic T lymphocytes in cancer immunotherapy: a review. J. Cell. Physiol. *234*, 8509–8521.

30. Trimble, C.L., Morrow, M.P., Kraynyak, K.A., Shen, X., Dallas, M., Yan, J., Edwards, L., Parker, R.L., Denny, L., Giffear, M., et al. (2015). Safety, efficacy, and immunogenicity of VGX-3100, a therapeutic synthetic DNA vaccine targeting human papillomavirus 16 and 18 E6 and E7 proteins for cervical intraepithelial neoplasia 2/3: a randomised, double-blind, placebo-controlled phase 2b trial. Lancet *386*, 2078–2088.

31. Morrow, M.P., Kraynyak, K.A., Sylvester, A.J., Shen, X., Amante, D., Sakata, L., Parker, L., Yan, J., Boyer, J., Roh, C., et al. (2016). Augmentation of cellular and humoral immune responses to HPV16 and HPV18 E6 and E7 antigens by VGX-3100. Mol. Ther. Oncolytics *3*, 16025.

32. Morrow, M.P., Kraynyak, K.A., Sylvester, A.J., Dallas, M., Knoblock, D., Boyer, J.D., Yan, J., Vang, R., Khan, A.S., Humeau, L., et al. (2018). Clinical and immunologic biomarkers for histologic regression of high-grade cervical dysplasia and clearance of HPV16 and HPV18 after immunotherapy. Clin. Cancer Res. *24*, 276–294.

33. Aggarwal, C., Cohen, R.B., Morrow, M.P., Kraynyak, K.A., Sylvester, A.J., Knoblock, D.M., Bauml, J.M., Weinstein, G.S., Lin, A., Boyer, J., et al. (2019). Immunotherapy targeting HPV16/18 generates potent immune responses in HPV-associated head and neck cancer. Clin. Cancer Res. *25*, 110–124.

34. Pasini, E., Caggiari, L., Dal Maso, L., Martorelli, D., Guidoboni, M., Vaccher, E., Barzan, L., Franchin, G., Gloghini, A., De Re, V., et al. (2009). Undifferentiated nasopharyngeal carcinoma from a nonendemic area: protective role of HLA allele products presenting conserved EBV epitopes. Int. J. Cancer *125*, 1358–1364.

35. Lin, K.Y., Guarnieri, F.G., Staveley-O'Carroll, K.F., Levitsky, H.I., August, J.T., Pardoll, D.M., and Wu, T.C. (1996). Treatment of established tumors with a novel vaccine that enhances major histocompatibility class II presentation of tumor antigen. Cancer Res. *56*, 21–26.

36. Yan, J., Reichenbach, D.K., Corbitt, N., Hokey, D.A., Ramanathan, M.P., McKinney, K.A., Weiner, D.B., and Sewell, D. (2009). Induction of antitumor immunity in vivo following delivery of a novel HPV-16 DNA vaccine encoding an E6/E7 fusion antigen. Vaccine *27*, 431–440.

37. Villarreal, D.O., Wise, M.C., Walters, J.N., Reuschel, E.L., Choi, M.J., Obeng-Adjei, N., Yan, J., Morrow, M.P., and Weiner, D.B. (2014). Alarmin IL-33 acts as an immunoadjuvant to enhance antigen-specific tumor immunity. Cancer Res. *74*, 1789–1800.

38. Suschak, J.J., Williams, J.A., and Schmaljohn, C.S. (2017). Advancements in DNA vaccine vectors, non-mechanical delivery methods, and molecular adjuvants to increase immunogenicity. Hum. Vaccin. Immunother. *13*, 2837–2848.

39. Smith, T.R.F., Patel, A., Ramos, S., Elwood, D., Zhu, X., Yan, J., Gary, E.N., Walker, S.N., Schultheis, K., Purwar, M., et al. (2020). Immunogenicity of a DNA vaccine candidate for COVID-19. Nat. Commun. *11*, 2601.

40. Gary, E.N., Warner, B.M., Parzych, E.M., Griffin, B.D., Zhu, X., Tailor, N., Tursi, N.J., Chan, M., Purwar, M., Vendramelli, R., et al. (2021). A novel mouse AAV6 hACE2 transduction model of wild-type SARS-CoV-2 infection studied using synDNA immunogens. iScience *24*, 102699.

41. Hutnick, N.A., Myles, D.J., Bian, C.B., Muthumani, K., and Weiner, D.B. (2011). Selected approaches for increasing HIV DNA vaccine immunogenicity in vivo. Curr. Opin. Virol. *1*, 233–240.

42. Khoshnejad, M., Dia, Y., Patel, A., Xu, Z., Zhu, X., Yun, K., Wojtak, K., Qureshi, R., Humeau, L., Muthumani, K., et al. (2020). DNA-encoded glutamine synthetase enzyme as ammonia-lowering therapeutic for hyperammonemia. Nucleic Acid Ther. *30*, 379–391.

43. Taylor, G.S., Haigh, T.A., Gudgeon, N.H., Phelps, R.J., Lee, S.P., Steven, N.M., and Rickinson, A.B. (2004). Dual stimulation of Epstein-Barr Virus (EBV)-specific CD4+- and CD8+-T-cell responses by a chimeric antigen construct: potential therapeutic vaccine for EBV-positive nasopharyngeal carcinoma. J. Virol. *78*, 768–778.

44. Lin, M.C., Lin, Y.C., Chen, S.T., Young, T.H., and Lou, P.J. (2017). Therapeutic vaccine targeting Epstein-Barr virus latent protein, LMP1, suppresses LMP1-expressing tumor growth and metastasis in vivo. BMC Cancer *17*, 18.

45. Duperret, E.K., Wise, M.C., Trautz, A., Villarreal, D.O., Ferraro, B., Walters, J., Yan, J., Khan, A., Masteller, E., Humeau, L., et al. (2018). Synergy of immune checkpoint blockade with a novel synthetic consensus DNA vaccine targeting TERT. Mol. Ther. *26*, 435–445.

46. Karyampudi, L., Lamichhane, P., Scheid, A.D., Kalli, K.R., Shreeder, B., Krempski, J.W., Behrens, M.D., and Knutson, K.L. (2014). Accumulation of memory precursor CD8 T cells in regressing tumors following combination therapy with vaccine and anti-PD-1 antibody. Cancer Res. *74*, 2974–2985.

47. Soares, K.C., Rucki, A.A., Wu, A.A., Olino, K., Xiao, Q., Chai, Y., Wamwea, A., Bigelow, E., Lutz, E., Liu, L., et al. (2015). PD-1/PD-L1 blockade together with vaccine therapy facilitates effector T-cell infiltration into pancreatic tumors. J. Immunother. *38*, 1–11.

48. Perales-Puchalt, A., Duperret, E.K., Yang, X., Hernandez, P., Wojtak, K., Zhu, X., Jung, S.H., Tello-Ruiz, E., Wise, M.C., Montaner, L.J., et al. (2019). DNA-encoded bispecific T cell engagers and antibodies present long-term antitumor activity. JCI Insight *4*, e126086.

49. Hoebe, E., Wille, C., Hagemeier, S., Kenney, S., Greijer, A., and Middeldorp, J. (2018). Epstein-Barr virus gene BARF1 expression is regulated by the epithelial differentiation factor DeltaNp63alpha in undifferentiated nasopharyngeal carcinoma. Cancers (Basel) *10*, 76.

OMTO, Volume *24*

Supplemental information

DNA immunotherapy targeting BARF1 induces potent anti-tumor responses against Epstein-Barr-virus-associated carcinomas

Xizhou Zhu, Alfredo Perales-Puchalt, Krzysztof Wojtak, Ziyang Xu, Kun Yun, Pratik S. Bhojnagarwala, Devivasha Bordoloi, Daniel H. Park, Kevin Liaw, Mamadou A. Bah, Paul M. Lieberman, Ebony N. Gary, Ami Patel, and David B. Weiner

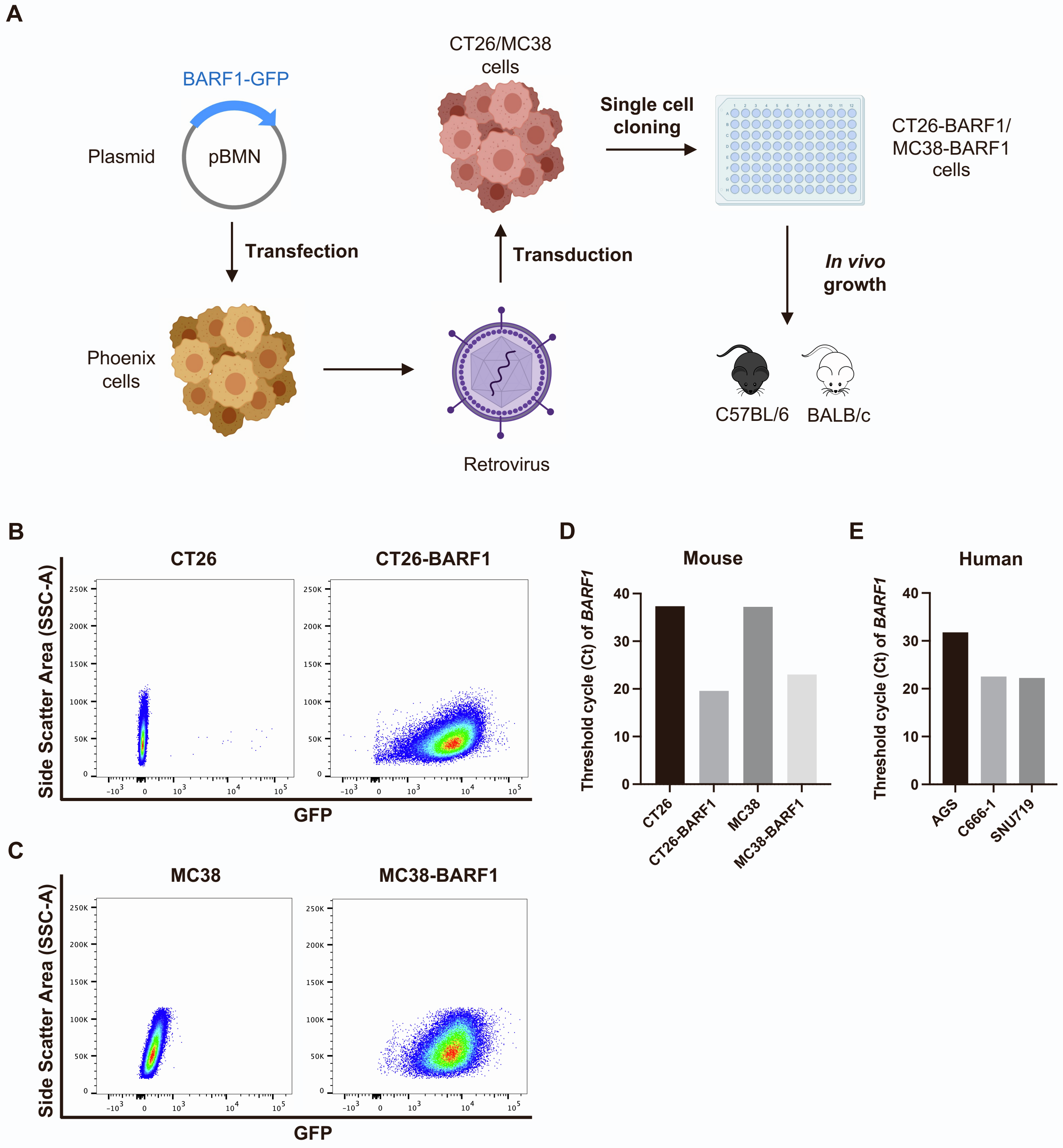

Supplemental Figure 1. Generation and validation of BARF1⁺ tumor models. (A) Workflow of retroviral transduction and single-cell cloning of CT26-BARF1 and MC38-BARF1 cells. (B and C) Clonality of CT26-BARF1 (B) and MC38-BARF1 (C) cell lines, after single-cell cloning, shown by GFP expression through flow cytometry. (D and E) Threshold cycles of *BARF1* gene amplification by RT-qPCR in transduced and parental mouse cancer cell lines (D) and human EBV positive (C666-1 and SUN719) and negative (AGS) cancer cell lines (E).

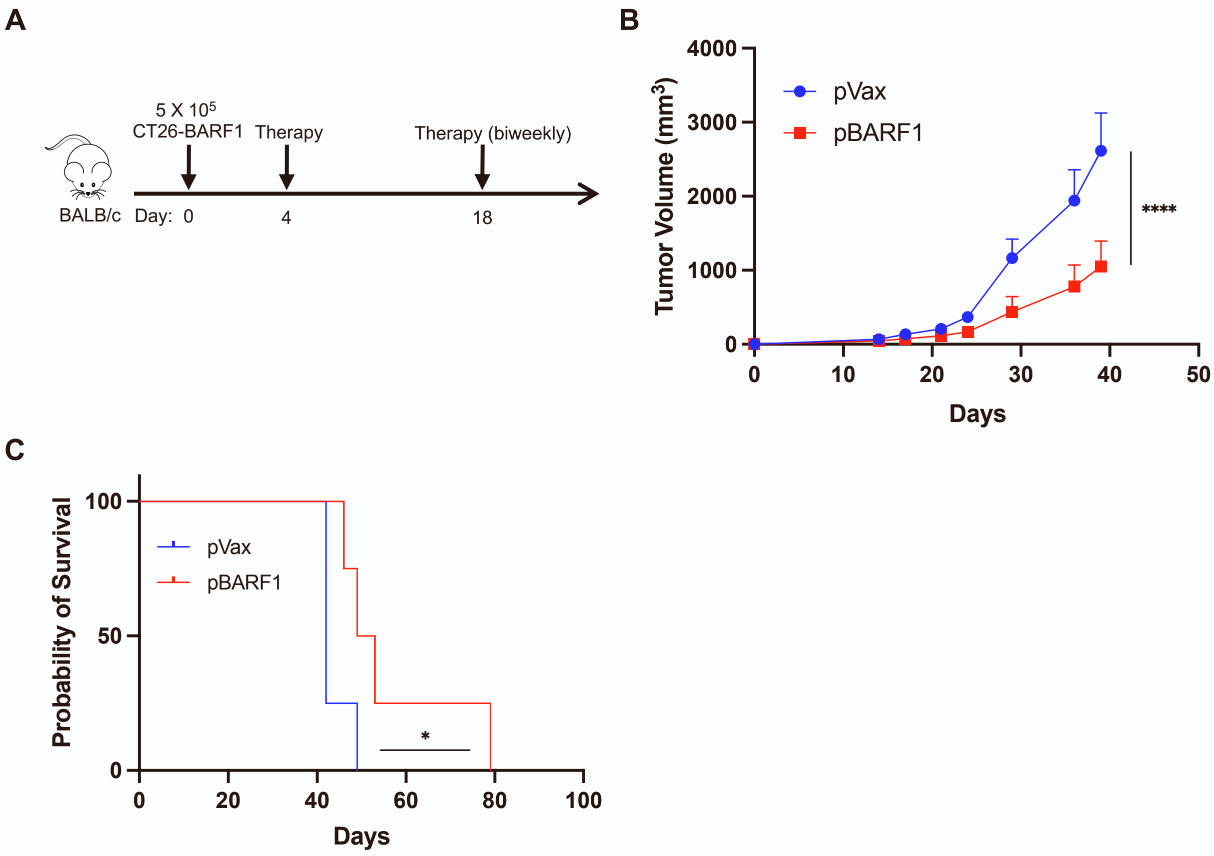

Supplemental Figure 2. pBARF1 improves survival in the therapeutic tumor model in BALB/c mice. (A) Study outline for the therapeutic tumor model. The mice were injected with CT26-BARF1 cells and immunized with pBARF1 biweekly starting on day 4. (B) Tumor volume measurements of the study described in (A). (C) Survival curve of the study described in (A). Significance for tumor volume was determined by two-way ANOVA. Significance for survival was determined by the log-rank test. *$p < 0.05$, ****$p < 0.0001$. n = 4 mice/group. Error bars indicate mean ± SEM.

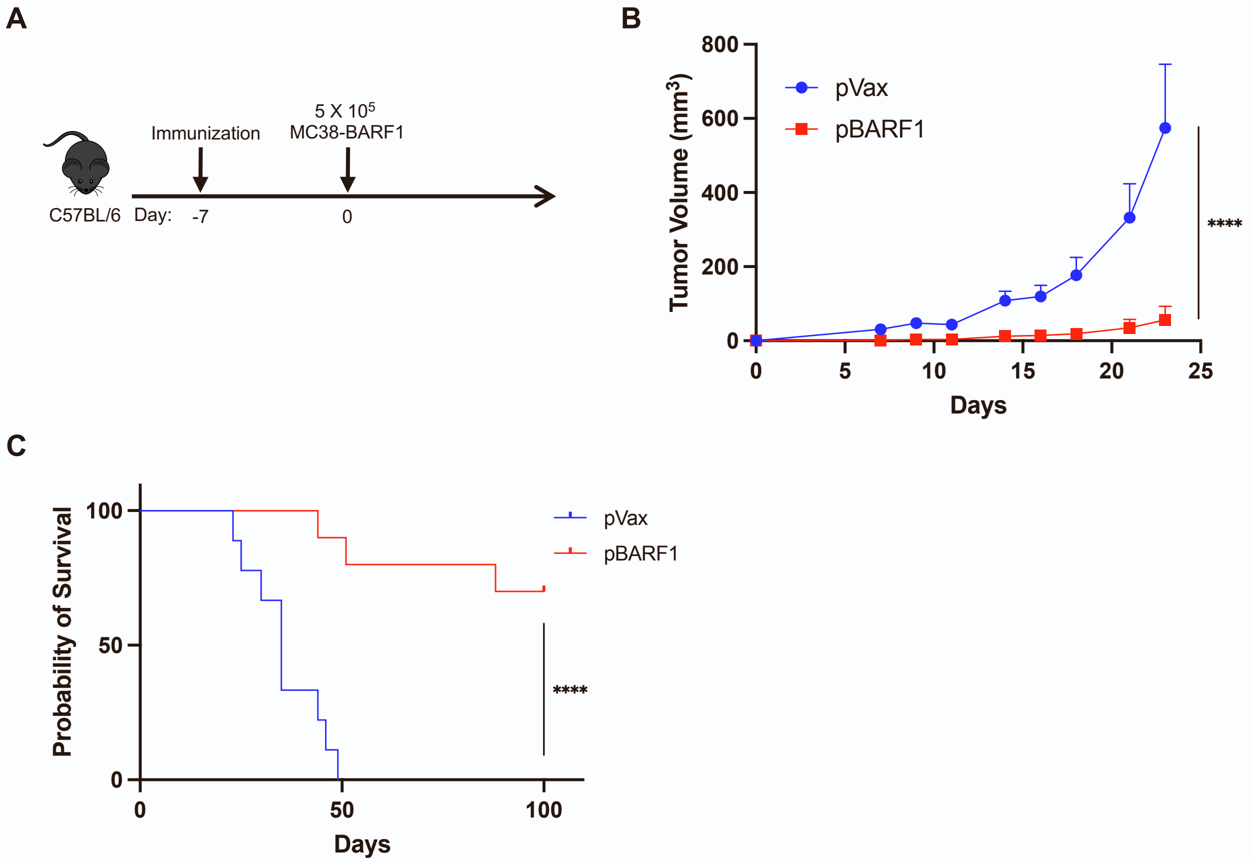

Supplemental Figure 3. Single immunization of pBARF1 prevents tumor progression in C57BL/6 mice. (A) Study outline for the pre-challenge immunization model. The mice were immunized with one dose of pBARF1 and injected with MC38-BARF1 cells one week after immunization. (B) Tumor volume measurements of the study described in (A). (C) Survival curve of the study described in (A). Significance for tumor volume was determined by two-way ANOVA. Significance for survival was determined by the log-rank test. ****$p < 0.0001$. $n = 10$ mice/group. Error bars indicate mean ± SEM.
